# Missing Data in Orthopaedic Clinical Outcomes Research: A Sensitivity Analysis of Imputation Techniques Utilizing a Large Multicenter Total Shoulder Arthroplasty Database

**DOI:** 10.3390/jcm14113829

**Published:** 2025-05-29

**Authors:** Kevin A. Hao, Terrie Vasilopoulos, Josie Elwell, Christopher P. Roche, Keegan M. Hones, Jonathan O. Wright, Joseph J. King, Thomas W. Wright, Ryan W. Simovitch, Bradley S. Schoch

**Affiliations:** 1 Department of Orthopaedic Surgery & Sports Medicine, University of Florida, Gainesville, FL 32611, USA; 2 Department of Anesthesiology, University of Florida, Gainesville, FL 32611, USA; 3 Exactech, Inc., Gainesville, FL 32653, USAchris.roche@exac.com (C.P.R.); 4 Hospital for Special Surgery Florida, West Palm Beach, FL 33401, USA; 5 Department of Orthopaedic Surgery and Sports Medicine, Mayo Clinic, Jacksonville, FL 32224, USA

**Keywords:** missing data, multiple imputation, predictive mean matching, database, clinical outcome, patient outcome, joint replacement, arthroplasty

## Abstract

**Background:** When missing data are present in clinical outcomes studies, complete-case analysis (CCA) is often performed, whereby patients with missing data are excluded. While simple, CCA analysis may impart selection bias and reduce statistical power, leading to erroneous statistical results in some cases. However, there exist more rigorous statistical approaches, such as single and multiple imputation, which approximate the associations that would have been present in a full dataset and preserve the study’s power. The purpose of this study is to evaluate how statistical results differ when performed after CCA analysis versus imputation methods. **Methods:** This simulation study analyzed a sample dataset consisting of 2204 shoulders, with complete datapoints from a larger multicenter total shoulder arthroplasty database. From the sampled dataset of demographics, surgical characteristics, and clinical outcomes, we created five test datasets, ranging from 100 to 2000 shoulders, and simulated 10–50% missingness in the postoperative American Shoulder and Elbow Surgeons (ASES) score and range of motion in four planes in missing completely at random (MCAR), missing at random (MAR), and not missing at random (NMAR) patterns. Missingness in outcomes was remedied using CCA, three single imputation techniques, and two multiple imputation techniques. The imputation performance was evaluated relative to the native complete dataset using the root mean squared error (RMSE) and the mean absolute percentage error (MAPE). We also compared the mean and standard deviation (SD) of the postoperative ASES score and the results of multivariable linear and logistic regression to understand the effects of imputation on the study results. **Results:** The average overall RMSE and MAPE were similar for MCAR (22.6 and 27.2%) and MAR (19.2 and 17.7%) missingness patterns, but were substantially poorer for NMAR (37.5 and 79.2%); the sample size and the percentage of data missingness minimally affected RMSE and MAPE. Aggregated mean postoperative ASES scores were within 5% of the true value when missing data were remedied with CCA, and all candidate imputation methods for nearly all ranges of sample size and data missingness when data were MCAR or MAR, but not when data were NMAR. When data were MAR, CCA resulted in overestimates of the SD. When data were MCAR or MAR, the accuracy of the regression estimate (β or OR) and its corresponding 95% CI varied substantially based on the sample size and proportion of missing data for multivariable linear regression, but not logistic regression. When data were MAR, the width of the 95% CI was up to 300% larger when CCA was used, whereas most imputation methods maintained the width of the 95% CI within 50% of the true value. Single imputation with k-nearest neighbor (kNN) method and multiple imputation with predictive mean matching (MICE-PMM) best-reproduced point estimates and intervariable relationships resembling the native dataset. Availability of correlated outcome scores improved the RMSE, MAPE, accuracy of the mean postoperative ASES score, and multivariable linear regression model estimates. **Conclusions:** Complete-case analysis can introduce selection bias when data are MAR, and it results in loss of statistical power, resulting in loss of precision (i.e., expansion of the 95% CI) and predisposition to false-negative findings. Our data demonstrate that imputation can reliably reproduce missing clinical data and generate accurate population estimates that closely resemble results derived from native primary shoulder arthroplasty datasets (i.e., prior to simulated data missingness). Further study of the use of imputation in clinical database research is critical, as the use of CCA may lead to different conclusions in comparison to more rigorous imputation approaches.

## 1. Introduction

Thousands of orthopaedic surgery studies have relied on large joint replacement registries to answer clinical question over the last decade, without robust measures for ensuring data completeness, which can vary amongst databases. Large registries play an important role in advancing clinical practice by providing researchers with the necessary sample sizes to study rare complications (e.g., postoperative acromion and scapular spine fractures), facilitating the identification of subtle differences in clinical outcomes, and providing information that is generalizable to patients across a larger geographic region. However, large registries have been (and will continue to be) limited by missing data. Commonly, researchers have handled missing data with complete-case analysis (CCA), in which patients with key missing data are excluded. However, this method of treating missing values is subject to selection bias, and thus it may be inferior to more rigorous statistical approaches such as imputation, which approximates the associations that would have been present in a full dataset [1,2]. Although imputation for missing data is routinely used in medical research overall, it has only recently begun to garner interest in orthopaedic research. One study of hip and knee arthroplasty suggested that the results obtained using data handled with CCA versus multiple imputation methods may produce differing results [3]. However, the influence of factors such as the imputation method, sample size, and the proportion of missing data on estimates obtained from imputation remains unknown and makes its adoption by researchers challenging.

The purpose of this simulation study is to determine whether the use of imputation to remedy missing data enables the generation of study data that more closely resemble the native dataset compared to complete-case analysis for varying (1) cohort sizes, (2) patterns of data missingness, (3) proportions of missing data, (4) types of imputation methods, and (5) statistical tests employed. Primarily, we performed a sensitivity analysis for a single validated patient-reported outcome measure, the American Shoulder and Elbow Surgeons (ASES) score. Secondarily, we performed this analysis on shoulder range of motion in forward elevation. Our hypotheses were three-fold, described as follows: (1) imputation would enable accurate reproduction of point estimates of missing outcome data, (2) common statistical analyses performed on imputed data would more closely resemble analyses performed on the native, complete dataset compared to complete case analysis, and (3) the accuracy of imputed data and subsequent analyses would depend upon data missingness characteristics.

## 2. Materials and Methods

### 2.1. Data Source

After institutional review board approval, we retrospectively sampled a prospectively-collected multicenter international shoulder arthroplasty database of a single platform prosthesis (Equinoxe, Exactech Inc., Gainesville, FL, USA) to create a sample dataset for this analysis. Patients undergoing primary anatomic TSA (aTSA) or reverse TSA (rTSA) between 2013 and 2021 at one of 40 distinct sites were eligible. We excluded patients with a preoperative diagnosis of revision TSA, fracture, post-traumatic arthritis, or infection. Patients were also excluded if they underwent reoperation within 2 years of surgery, developed a postoperative periprosthetic joint infection, postoperative nerve injury, or dislocation. To evaluate the performance of the strategies used to remedy missing data, we required a ground truth dataset without missing values; thus, we extracted a sampling of patients with complete preoperative and postoperative data of interest and a follow-up greater than 2 years. This sample cohort consisted of 2204 TSAs (795 aTSAs, 1409 rTSAs).

### 2.2. Clinical Outcome Measures

To maximize the generalizability of this study, we chose to evaluate the American Shoulder and Elbow Surgeons (ASES) score and shoulder range of motion in active (ROM) forward elevation (FE) [4,5]. The ASES score and ROM were evaluated pre- and postoperatively at each follow-up visit. Range of motion in active FE was obtained by the operating surgeon or surrogate research coordinator using a standardized protocol across clinical sites. In addition to the ASES score, the Constant score and the Shoulder Arthroplasty Smart (SAS) score were also collected to determine how the presence of correlated outcomes influences the results of imputation [6,7].

### 2.3. Demographic and Surgical Covariates

Patient and surgical characteristics hypothesized to provide prognostic information were identified for consideration of inclusion into imputation models. Patient demographic characteristics included age at surgery, sex, whether the operative side was their dominant shoulder, whether the patient had a history of prior surgery on the involved shoulder, the presence of medical comorbidities, and the preoperative diagnosis for their TSA. Comorbidities evaluated included inflammatory arthritis, hypertension, heart disease, diabetes mellitus, and tobacco use. Preoperative shoulder diagnoses included osteoarthritis (OA), osteonecrosis (ON), rotator cuff tear (RCT), rotator cuff tear arthropathy (RCA), and rheumatoid arthritis (RA). Surgical variables included the type of prosthesis received (aTSA vs. rTSA), whether the subscapularis was repaired, whether a cemented stem was used, and the intraoperative estimated blood loss.

### 2.4. Introduction of Data Missingness

**Figure 1:**
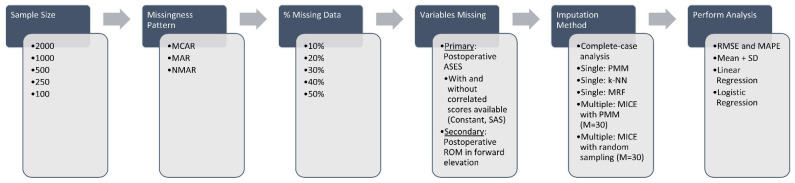
Simulation framework. *ASES*, American Shoulder and Elbow Surgeons; *k-NN*, k-nearest neighbor; *MAPE*, mean absolute percentage error; *MAR*, missing at random; *MCAR*, missing completely at random; *MICE*, multiple imputation with chained equations; *MRF*, multivariate random forest; *NMAR*, not missing at random; *PMM*, predictive mean matching; *RMSE*, root mean squared error; *ROM*, range of motion; *SAS*, shoulder arthroplasty smart; *SD*, standard deviation.

Our simulation framework is depicted in Figure 1. First, random subsamples of the sample dataset (n = 2204) were created. We chose to evaluate sample sizes of 2000, 1000, 500, 250, and 100 shoulders, as this effectively covers the range of sample sizes in most orthopaedic clinical database studies. Next, we created missing data using the following missing data mechanisms: missing completely at random (MCAR), missing at random (MAR), and not missing at random (NMAR). The proportions of missing data evaluated were 10%, 20%, 30%, 40%, and 50%. The mechanisms of missing data are reviewed briefly in Table 1. Towards our primary aim, MCAR was simulated by randomly deleting postoperative ASES scores using a random number generator in R. MAR was simulated by disproportionately deleting postoperative ASES scores in patients with high preoperative ASES scores, as previous studies have demonstrated that pre- and postoperative ASES scores are strongly correlated [8,9,10]. Finally, NMAR was simulated by disproportionally deleting the lowest postoperative ASES scores. An example of how these mechanisms were applied is provided in Table 2.

Towards our secondary aim, these missingness patterns were also simulated for ROM. We implemented MCAR, MAR, and NMAR patterns based on the values for FE given its importance to shoulder function and the prevalence of evaluation. In clinical practice, patients that are prospectively followed either have ROM in all planes evaluated at a given clinical visit or they have none evaluated; therefore, shoulders with deleted FE values also had ROM in the remaining three planes deleted.

### 2.5. Imputation Strategies

After data missingness patterns were created in random samples, we evaluated five candidate imputation strategies, encompassing single imputation strategies and multiple imputation strategies. For comparison, CCA was also evaluated. Single imputation strategies included predictive mean matching (PMM) [11], k-nearest neighbor (kNN) [11], and multivariate random forest (MRF) algorithms [12]. Multiple imputation strategies included multivariate imputation by chained equations using PMM and random sampling techniques performed with 30 multiple imputations (M). These methods are briefly described in Table 3. Single imputation was performed using the *simputation* package [12] and multiple imputation was performed using the *mice* package [13]. Imputation models for the ASES score were informed by the preoperative (all shoulders) and postoperative (for shoulders without missing values) ASES score and all demographic and surgical characteristics described previously. Similarly, imputation models for ROM were informed by preoperative and postoperative ROM and all demographic and surgical characteristics. Imputation models for the ASES score were not informed by ROM and vice versa.

### 2.6. Performance Assessment

The ability of imputation techniques to reproduce the original data and intervariable relationships for the ASES score and ROM in each plane was evaluated by assessing the point estimate error, population-level statistics, and regression estimates. First, each imputation method’s performance on estimating point values was quantified by calculating the root mean square error (RMSE) and mean absolute percentage error (MAPE) relative to the true values (zero denoting perfect reproduction of native data). The RMSE provides a broad representation of the error distribution from the method/model, whereas the MAPE gives an intuitive interpretation for the relative error. Second, the absolute value of the percent error for the mean and standard deviation (SD) of each imputed outcome measure relative to the true values was calculated and plotted for a visual comparison of the distribution between imputation techniques. Third, we evaluated how imputation influenced estimates of the association between pre- and postoperative outcome measures using multivariable linear and logistic regression models, controlling for age, sex, BMI, history of prior surgery, and type of shoulder arthroplasty. Linear regression results were represented by the β coefficient and the corresponding 95% confidence interval (CI), denoting the change in the postoperative outcome measure with every one-unit change in the preoperative outcome measure. The logistic regression results were represented with the odds ratio (OR) and corresponding 95% CI denoting the increase in odds of achieving a 90th percentile outcome associated with each one-unit change in the preoperative outcome measure. The 90th percentile for each outcome measure was calculated for the overall sample cohort (n = 2204), as follows: ASES score = 100 points, FE = 170°, abduction = 160°, ER = 70°, and IR score = 6 points (i.e., T12–T8). To compare estimates (β and OR) and the width of associated 95% CIs generated from imputed and CCA datasets relative to the overall sample datasets, the absolute percent error was calculated and plotted. All data analyses were performed in R Software (version 4.2.0, R Core Team, Vienna, Austria).

## 3. Results

### 3.1. Patient Characteristics

The sample study dataset comprised 2204 shoulders, including 795 aTSAs and 1409 rTSAs (Table 4). The mean age at surgery was 69 years old, 56% of shoulders were female, and the mean follow-up was 30 months. The predominant preoperative diagnosis of aTSAs was primary osteoarthritis (95%), whereas preoperative diagnoses in rTSAs were most commonly osteoarthritis (49%) and rotator cuff tear arthropathy (35%). The subscapularis was repaired in 38% of rTSAs. The ASES score and ROM in all planes improved pre- to postoperatively (*p* < 0.001). On average, the ASES score improved from 38.6 ± 16.0 preoperatively to 84.4 ± 17.6 postoperatively.

### 3.2. ASES Score

**Figure 2:**
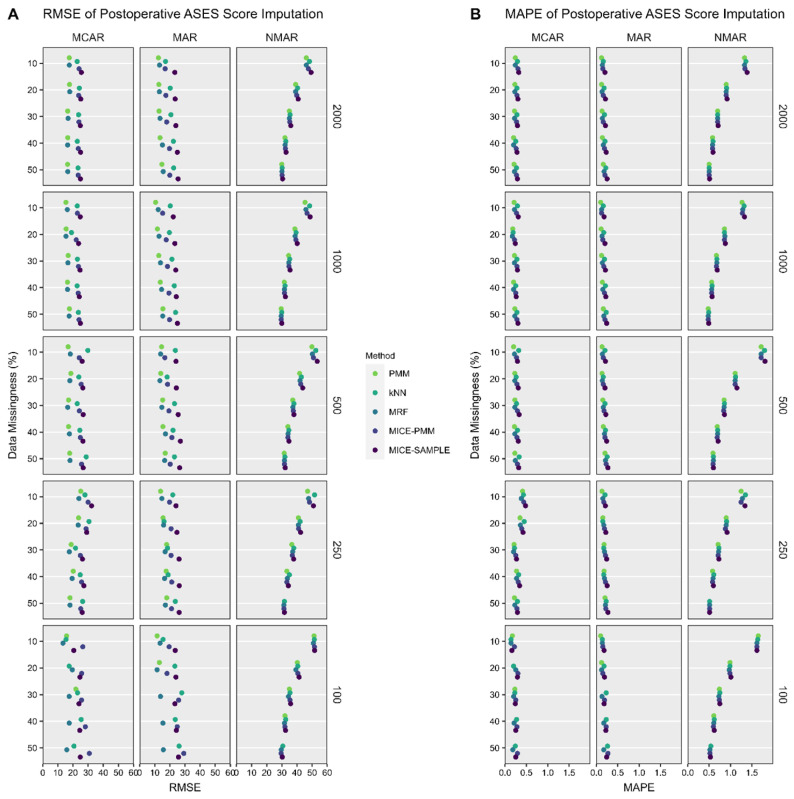
RMSE (**A**) and MAPE (**B**) (1.0 = 100%) denoting the error between imputed values and the original native data that were deleted. Single imputation with PMM and MRF and MICE with PMM performed well for MCAR and MAR conditions, whereas all methods performed poorly when data were NMAR. *ASES*, American Shoulder and Elbow Surgeons; *k-NN*, k-nearest neighbor; *MAPE*, mean absolute percentage error; *MAR*, missing at random; *MCAR*, missing completely at random; *MICE*, multiple imputation with chained equations; *MRF*, multivariate random forest; *NMAR*, not missing at random; *PMM*, predictive mean matching; *RMSE*, root mean squared error.

All candidate imputation methods successfully imputed missing values across all of the sample sizes, with the exception of single imputation PMM, which failed to impute missing values in the dataset with 100 shoulders when >10% of the data were missing. The average overall RMSE and MAPE were similar for the MCAR (23% and 27%) and MAR (19% and 18%) missingness patterns, but were substantially poorer for NMAR (38% and 79%) (Figure 2). In general, single imputation PMM and MRF generated more accurate estimates of the postoperative ASES score compared to other candidate methods, although these differences were minimal. The sample size and the percentage of data missingness did not influence the performance of imputation methods when evaluated with the RMSE and MAPE.

**Figure 3:**
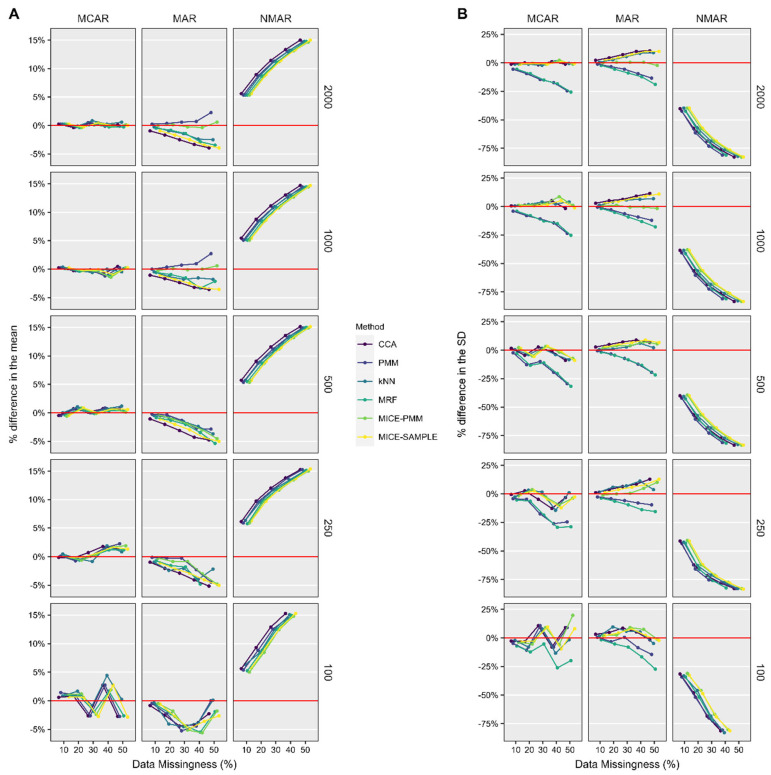
Percent difference in the mean (**A**) and standard deviation (**B**) of postoperative ASES scores after missing data were remedied by each of the candidate methods compared to the native, complete sample dataset. *ASES*, American Shoulder and Elbow Surgeons; *k-NN*, k-nearest neighbor; *MAR*, missing at random; *MCAR*, missing completely at random; *MICE*, multiple imputation with chained equations; *MRF*, multivariate random forest; *NMAR*, not missing at random; *PMM*, predictive mean matching; *SD*, standard deviation.

Estimates of the mean postoperative ASES score were within 5% of the true value when the missing data were remedied with CCA and all candidate imputation methods for nearly all ranges of sample size and data missingness, when data were MCAR or MAR, but not when data were NMAR (Figure 3). When data were MCAR and NMAR, imputation offered little improvement over CCA in the accuracy of estimates of the mean and SD. However, when data were MAR, CCA resulted in overestimates of the SD, whereas single imputation PMM and MRF underestimated the SD. MICE-PMM generated estimates of the mean and SD that most closely resembled the native dataset; this was particularly evident for large sample sizes (i.e., 1000 and 2000 shoulders), but still outperformed CCA at lower sample sizes.

**Figure 4:**
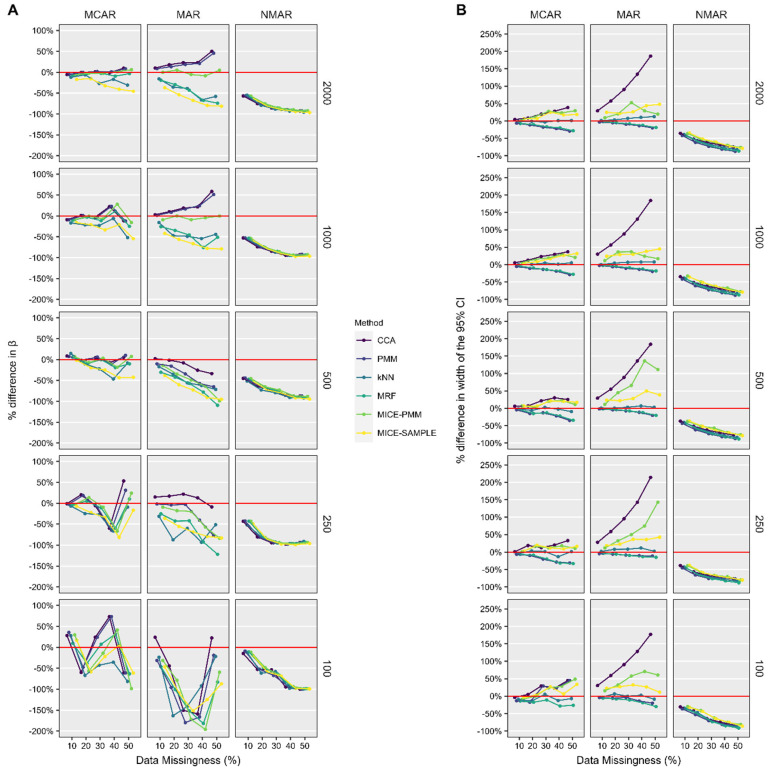
Percent difference in the regression coefficient β (**A**) and the width of the 95% CI (**B**) for the relationship between preoperative and postoperative ASES score from multivariable linear regression performed on datasets after candidate methods were utilized to remedy missing data compared to the native, complete dataset. *ASES*, American Shoulder and Elbow Surgeons; *CI*, confidence interval; *k-NN*, k-nearest neighbor; *MAR*, missing at random; *MCAR*, missing completely at random; *MICE*, multiple imputation with chained equations; *MRF*, multivariate random forest; *NMAR*, not missing at random; *PMM*, predictive mean matching.

In the multivariable linear regression analysis (Figure 4), the accuracy of the estimate β and its corresponding 95% CI varied substantially based on the sample size and proportion of missing data. When data were MCAR and MAR, estimations of β could be accurately performed when up to 10% of data were missing with 100 samples, 30% with 250 samples, and up to 50% with 500 or more samples. The width of the 95% CI was within 50% of the true value for CCA and all imputation methods when data were MCAR. However, when data were MAR, the width of the 95% CI was nearly 200% larger when CCA was used, whereas most imputation methods kept it within 50% of the true value. Overall, the most accurate estimates of β and its corresponding 95% CI were generated when the missing data were imputed with k-NN single imputation and MICE-PMM (particularly with 1000 or more shoulders), respectively.

**Figure 5:**
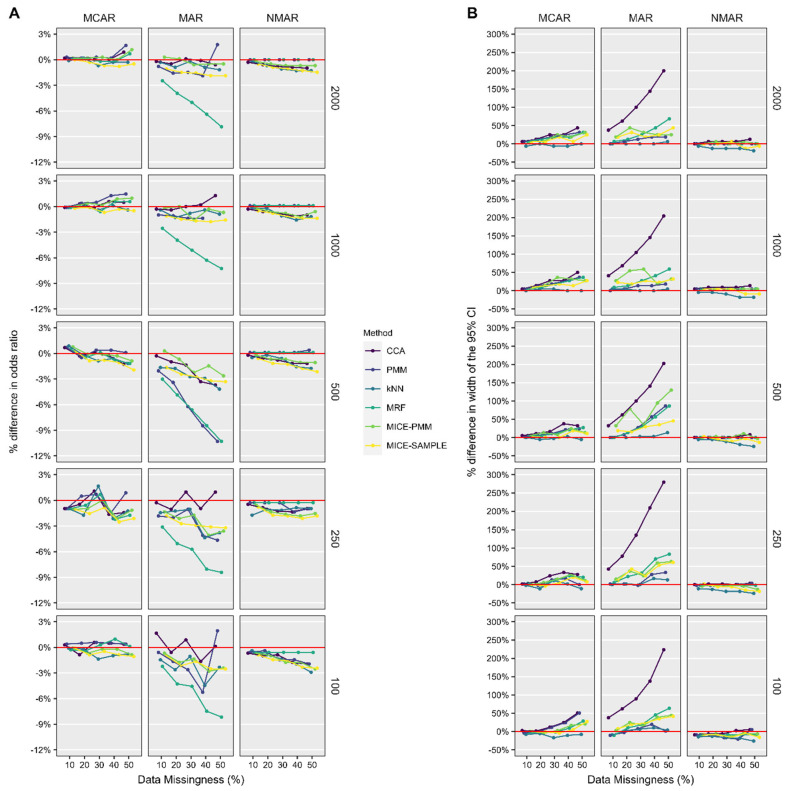
Percent difference in the odds ratio (**A**) and the width of the 95% CI (**B**) for obtaining a 90th percentile ASES score from multivariable logistic regression performed on datasets after candidate methods were utilized to remedy missing data compared to the native, complete dataset. *ASES*, American Shoulder and Elbow Surgeons; *CI*, confidence interval; *k-NN*, k-nearest neighbor; *MAR*, missing at random; *MCAR*, missing completely at random; *MICE*, multiple imputation with chained equations; *MRF*, multivariate random forest; *NMAR*, not missing at random; *PMM*, predictive mean matching.

In the multivariable logistic regression analysis (Figure 5), the accuracy of the odds ratio did not vary substantially based on the sample size and proportion of missing data, with nearly all estimates being within 5% of the true value. When data were MAR, single imputation with MRF resulted in a substantially reduced odds ratios for all sample sizes and proportions of missing data. Similar to the multivariable linear regression results, the width of the 95% CI was up to 300% larger when CCA was used, whereas most imputation methods kept it within 50% of the true value. Overall, the most accurate estimates of the odds ratio and its corresponding 95% CI were generated when missing data were imputed with k-NN single imputation.

### 3.3. ASES Score Imputed with Correlated Scores Available

The availability of postoperative Constant and SAS scores improved the RMSE and MAPE of imputed ASES scores across all methods (Supplementary Figure S1); MICE-PMM also performed similarly to single imputation PMM and MRF. Estimates of the mean postoperative ASES score improved from within 5% to within 2% of the true value for all methods when data were MCAR or MAR, with the best performance found for single imputation k-NN and MICE-PMM (Supplementary Figure S2). Estimates of the SD were similar when the ASES score was imputed with and without correlated scores available; in addition to single imputation k-NN, MICE-PMM also performed well. The β coefficient and its associated 95% CI, estimated from imputed data, were improved by the availability of correlated scores, particularly for low sample sizes and high proportions of missing data (Supplementary Figure S3). The availability of correlated scores led to a similar performance in all single imputation methods and MICE-PMM, but CCA and MICE-SAMPLE continued to perform poorly. The accuracy of the odds ratio and its associated 95% CI generated from imputation methods was similar with and without correlated scores being available (Supplementary Figure S4).

### 3.4. Forward Elevation

Imputed postoperative FE had similar RMSE and MAPE to imputed ASES scores in the primary analysis, with single imputation PMM and MRF performing the best (Supplementary Figure S5). Similar to estimates of the mean postoperative ASES score in the primary analysis, estimates of the mean postoperative FE were similarly within 5% of the true value for all methods when the data were MCAR or MAR, with the best performance found for single imputation k-NN and MICE-PMM (Supplementary Figure S6). The accuracy of the results of multivariable linear and logistic regression (β and odds ratio with their respective 95% CIs, respectively) evaluating the relationship between preoperative and postoperative FE mirrored the results of the primary analysis for the ASES score (Supplementary Figures S7 and S8), with the best performance found for single imputation k-NN and MICE-PMM.

## 4. Discussion

This simulation study is the first to evaluate how missing clinical data influence the results of commonly used statistical analyses. Our data confirm our hypotheses, which are as follows: (1) imputation enables accurate reproduction of point estimates of missing outcome data; (2) common statistical analyses performed on imputed data in most instances more closely resemble analyses performed on the native, complete dataset compared to CCA (and, notably, even in the MNAR simulation, for which imputation is not recommended, no imputation methods were notably worse than CCA); and (3) the accuracy of imputed data and subsequent analyses depend upon data missingness characteristics. This study contributes importantly to the advancement of clinical research because the generation of large datasets through subspecialty registries and multicenter collaborations are becoming increasingly common and are undoubtedly impacted by missing data. Our findings provide a basis to inform researchers on how to appropriately remedy missing data to mitigate potential selection biases and retain statistical power.

CCA is commonly used to handle missing data in clinical research, including orthopaedic studies. However, this approach is problematic as it results in a possible selection bias in reported outcomes and reduces statistical power. This is demonstrated in this study by the exorbitant increase in the width of the 95% CI when linear and logistic regression were applied to data MAR and by the less accurate means in the simulated MAR scenarios. Similar observations were made by Ondeck et al [3]., who used the National Surgical Quality Improvement Program database to evaluate the results of logistic regression when performed with CCA versus multiple imputation using a logistic regression model. The authors found that these two different methods of handling missing data led to differing associations of low preoperative laboratory values with adverse events after unicompartmental total knee arthroplasty. Our study builds upon this prior work in several important ways. Our study utilized a native, complete dataset, with which we compared the result of CCA and imputation. We also compared several different imputation methods with varying complexity to evaluate their performance across a wide range of missing data characteristics (10–50% missing data, all three missingness patterns, varying sample sizes).

Missing data can be present in several different patterns. While typically categorized for intellectual discussion as MCAR, MAR, or NMAR, the reality is that the missing data in clinical databases are likely a blend of two or more of these patterns. Expanding on the example provided in Table 1, ASES scores may be missing through all three mechanisms, as follows: clinic coordinators occasionally forgot to administer questionnaires (MCAR), females less frequently filled out questionnaires (MAR), and patients with low ASES scores more often sought treatment at a different facility (NMAR). While arguments can be made for which of these three mechanisms predominately influences a given database, this cannot be determined empirically. Therefore, researchers need to assume that all three mechanisms are responsible for missing data to varying degrees when considering how to deal with missing data.

Based on our data, when performing outcome analyses of large orthopedic databases or registries, we would recommend that some method of imputation should be used instead of CCA when the sample size is at least 100. Notably, even in our simulation of NMAR, the situation for which imputation is typically not recommended, CCA fared no better than any of the imputation methods tested, and performed worse when some correlated outcome scores were available. However, it is more difficult to provide a definitive recommendation on the optimal imputation method. In this study, single imputation k-NN and multiple imputation MICE with PMM generally yielded data that most closely resembled the native dataset. However, the optimal imputation method may vary based on variables not tested herein, such as specific imputation method parameters (e.g., the number of datasets *M* specified when using *MICE*), the type of data being imputed, and the available covariates. For example, when correlated outcome scores were made available to the model during imputation, the performance of MICE-PMM improved, which is logical, since this method imputes missing values based on available values from similar cases. However, complexity should be considered when choosing an imputation method. While standard statistical methods can be used to calculate summary statistics and perform analyses after single imputation methods such as k-NN are used, Rubin’s rules must be applied to calculate statistical estimates across all imputed datasets *M* when multiple imputation is used. We believe that the most important change to research practice is to use some form of imputation instead of CCA; the specific method of imputation is secondary. Thus, our current recommendation to researchers is to utilize either single imputation k-NN or MICE-PMM, depending on the characteristics of their data and the resources available (e.g., access to a statistician, software availability).

Many institutions that track patient outcomes collect numerous outcome scores, which are often highly correlated. Commonly collected shoulder outcome scores include the ASES score [4], Constant score [6], the Patient-Reported Outcomes Measurement Information System (PROMIS) score [14,15], the Shoulder Pain and Disability Index (SPADI) [16], the Simple Shoulder Test (SST) [17], the Single Assessment Numerical Evaluation (SANE) [18], the University of California, Los Angeles (UCLA) score [19], and the Visual Analog Scale for Pain (VAS) [20]. In essence, all of these scores measure patients’ shoulder pain and function. Many of these scores have overlapping questions that ask patients to rate their pain on a daily basis or their ability to perform activities of daily living. Therefore, it is expected (and has been demonstrated empirically) that these scores are highly correlated [21,22]. However, there are a variety of reasons why data might be missing for some, but not all, of these scores. For example, the Constant scores partially comprise strength measurements, which may not be obtained during a virtual visit. While some of these scores, such as the ASES score and SST, are calculated based on responses to multiple questions, other scores like the SANE and VAS are derived from the response of a single question. Some patients may only partially complete questionnaires, prohibiting calculation of their ASES and SST scores, but they may have responded to the questions necessary to calculate the SPADI, SANE, and VAS scores. In a hypothetical study where the primary outcome is the ASES score, the availability of the SPADI, SANE, and VAS scores for patients without ASES scores can be utilized in imputation models to approximate the ASES score, thus preserving the statistical power of the hypothetical study.

Imputation can also facilitate performing retrospective multicenter studies. A restriction to performing multicenter studies can be differences in outcome scores that are collected. While conversion equations have been published that allow interconversion of different outcomes scores [22], this does not account for differences in baseline patient characteristics (e.g., age, sex, prior surgery). Instead, imputation models that include a myriad of patient characteristics and outcome scores could be used to combine data for multiple centers, despite the heterogeneity of data.

While this study highlights the importance of advancing the use of statistical methods to improve orthopedic data quality, it must be considered within its limitations. While we simulated a large range of data missingness (10–50%) and sample sizes (100–2000), this does not encompass all research scenarios clinicians encounter. We evaluated data missingness patterns in isolation and did not attempt to create datasets with mixed patterns (e.g., 10% MCAR, 10% MAR, 5% NMAR), which are more likely to resemble real databases. While we utilized five common imputation methods (including multiple imputation), the methods evaluated are not exhaustive. It is important to note that many of the imputation methods utilized in this study generally do not meet underlying assumptions for use with NMAR data [23]; nevertheless, we chose to include this condition in our analysis because we felt that some missing data in clinical databases will be subject to NMAR patterns, and it would be remiss of us to ignore them. However, given this context, the NMAR results should be considered exploratory, and caution is warranted regarding its interpretation. This study was performed using a multi-institutional international shoulder arthroplasty database; while we believe our results are generalizable to other clinical databases, future studies are needed to confirm this. We evaluated imputation of a common PROM (i.e., the ASES score) and ROM in FE, but we did not impute a binary event such as the incidence of complications or need for revision surgery. While this analysis would be valuable and should be performed in the future, we believe it is less urgent because these outcomes are more consistently tracked and are less likely to be “missing”. While we evaluated FE, we did not evaluate ROM in other planes (i.e., external rotation, internal rotation, abduction) because it would have resulted in an inordinate amount of data; we believe that the results of FE (which were very similar to the ASES score results) can be generalized to ROM in other planes.

## 5. Conclusions

Despite the widespread use of complete-case analysis, this method can introduce selection bias and results in the loss of statistical power, resulting in loss of precision (i.e., expansion of the 95% CI), and predisposes studies to false-negative findings. Our data demonstrate that imputation can be reliably used to reproduce missing clinical data and generate accurate population estimates that closely resemble results derived from native datasets (i.e., prior to simulated data missingness) for MCAR and MAR conditions, but not for NMAR. Further study of the use of imputation in clinical database research is critical, as the use of CCA may lead to different conclusions in comparison to more rigorous imputation approaches.

## Figures and Tables

**Table 1:** Types of missing data.

Missingness Pattern	Definition and Example
Missing Completely At Random (MCAR)	No systematic differences between missing and observed values are present. For example, ASES scores may be missing because clinic coordinators forgot to administer questionnaires that particular day.
Missing At Random (MAR)	Systematic difference between missing and observed values can be explained by differences in observed data. For example, missing ASES scores may be lower than recorded ASES scores, but only because females were less likely than males to fill out the questionnaire. In this scenario, if considering only the subgroup of patients who are female, the assumption can be made that the missing data for females should be similar, and hence can be imputed from the collected data for females. Therefore, if sex is used as a predictive factor in the imputation model, imputation methods are appropriate for use with this dataset.
Not Missing At Random (NMAR)	Even after the observed data are considered, systematic differences remain between the missing and observed values. For example, patients with low ASES scores may be more likely to have missing values because they sought treatment at a different institution.

ASES, American Shoulder and Elbow Surgeons.

**Table 2:** Example depicting the three types of data missingness introduced. In the MCAR condition, ASES scores are discarded independent of patient sex. In the MAR condition, ASES scores are discarded based on the value of sex. In this example, all discarded ASES scores correspond to female patients. In the NMAR condition, ASES scores are discarded based on the actual ASES score. In this example, low ASES scores are discarded.

Patient #	Sex	Postoperative ASES Score	MCAR	MAR	NMAR
1	M	40	?	40	?
2	M	83	83	83	83
3	F	65	65	?	?
4	M	88	?	88	88
5	F	85	85	?	85
6	F	25	25	?	?
7	F	35	?	?	?
8	M	87	?	87	87
9	F	62	?	?	?
10	F	85	85	85	85

ASES, American Shoulder and Elbow Surgeons; F, female; M, male; MAR, missing at random; MCAR, missing completely at random; NMAR, not missing at random.

**Table 3:** Candidate methods for dealing with missing data.

Method	Definition	Advantages	Disadvantages
Complete-case analysis (CCA)	Only patients with complete information are analyzed. Patients with missing data are excluded from analysis.	- Simple - Preserves original data	- Loss of sample size (and therefore study power), increased risk of type 2 error - Will likely introduce bias if data are not MCAR
Single imputation			
Predictive mean matching (PMM)	Missing values are imputed by first predicting them using a regression model based on observed values of other variables, then replacing the missing value with a value from a donor pool of observed values that are closest to the predicted value.	- Utilizes available data to impute missing values - Preserves variability in the dataset - Can handle continuous and categorical datatypes	- Assumes similar patients have similar missing values - May be biased by outliers since it relies on identifying similar cases
k-Nearest Neighbor (kNN)	Missing values are imputed by averaging or interpolating values from the k most similar observations (nearest neighbors) based on other variables.	- Utilizes available data to impute missing values - Captures non-linear relationships between variables	- Sensitive to input parameters (k, distance metric)
Multivariate random forest (MRF)	Missing values are imputed by building a random forest model for each variable with missing values and imputing missing values by averaging predictions from multiple trees in the forest.	- Robust to non-linearity, interactions and missing data patterns	- May not perform well if the relationships between variables are too complex or sparse
Multiple imputation			
MICE with PMM, M = 30 (MICE-PMM-30)	Missing values are imputed using predictive mean matching within each iteration of the MICE algorithm.	- Similar to single imputation PMM, but account for uncertainty in imputation - Generally produces more plausible imputed values	- Similar to single imputation PMM, but with added computational complexity
MICE with random sampling, M = 30 (MICE-SAMPLE-30)	Missing values are imputed by drawing random samples from the predictive distribution within each iteration of the MICE algorithm.	- Relatively simple and computationally efficient - Retains some variability in imputed values	- Imputed values may not always be plausible or realistic (e.g., outside the expected range) - May not perform well with complex or non-linear relationships

MICE, multiple imputation with chained equations.

**Table 4:** Demographic, surgical characteristics, and pre- and postoperative outcome measure values from the sample cohort with complete data (n = 2204).

Variable	Combined (n = 2204)	aTSA (n = 795)	rTSA (n = 1409)
Age at surgery (years)	69.3 ± 8.0	65.4 ± 7.7	71.6 ± 7.3
BMI (kg/m^2^)	29.6 ± 6.2	30.6 ± 6.4	29.1 ± 6.0
Follow-up (months)	30.3 ± 12.3	31.3 ± 14.1	29.8 ± 11.1
Female sex	55.6% (1226)	49.3% (392)	59.2% (834)
Dominant side surgery	61.2% (1348)	57.9% (460)	63.0% (888)
Comorbidities			
None	29.2% (644)	35.1% (279)	25.9% (365)
Hypertension	57.0% (1256)	51.6% (410)	60.0% (846)
Heart disease	15.7% (346)	13.3% (106)	17.0% (240)
Diabetes	14.9% (328)	12.8% (102)	16.0% (226)
Tobacco use	8.1% (178)	8.9% (71)	7.6% (107)
Preoperative diagnosis			
Osteoarthritis	65.5% (1444)	94.6% (752)	49.1% (692)
Avascular necrosis	1.7% (38)	1.9% (15)	1.6% (23)
Rotator cuff tear	7.2% (159)	0.4% (3)	11.1% (156)
Cuff tear arthropathy	22.7% (500)	0.9% (7)	35.0% (493)
Rheumatoid arthritis	2.9% (63)	2.3% (18)	3.2% (45)
Subscapularis repair	-	-	37.8% (533)
Cemented humeral component	5.4% (118)	5.5% (44)	5.3% (74)
Previous surgery on shoulder	23.8% (524)	16.2% (129)	28.0% (395)
Estimated blood loss (mL)	200 (100–300)	200 (100–300)	160 (100–300)
Preoperative outcome measures			
ASES score	38.6 ± 16.0	38.9 ± 16.0	38.4 ± 16.1
Active FE (°)	93 ± 36	102 ± 32	88 ± 37
Postoperative outcome measures			
ASES score	84.4 ± 17.6	86.6 ± 17.3	83.1 ± 17.7
Active FE (°)	145 ± 24	149 ± 25	142 ± 24
Improvement in outcome measures			
ASES score	45.8 ± 20.9	47.7 ± 21.0	44.7 ± 20.8
Active FE (°)	51 ± 38	47 ± 35	53 ± 39

ASES, American Shoulder and Elbow Surgeons; aTSA, anatomic total shoulder arthroplasty; BMI, body mass index; FE, forward elevation; rTSA, reverse total shoulder arthroplasty. Values provided are available N and either mean ± standard deviation or % (N), as appropriate.

## Data Availability

The datasets presented in this article are not readily available because the data are part of an ongoing study. Requests to access the datasets should be directed to Christopher P. Roche.

## Supplementary Materials

The following supporting information can be downloaded at: https://www.mdpi.com/article/10.3390/jcm14113829/s1.
