# Supplementary material for: Missing Data in Orthopaedic Clinical Outcomes Research: A Sensitivity Analysis of Imputation Techniques Utilizing a Large Multicenter Total Shoulder Arthroplasty Database
Source: J Clin Med. 2025 May 29;14(11):3829. doi: 10.3390/jcm14113829 (PMC12157154; doi:10.3390/jcm14113829)
Supplement: Supplementary file 1 [file jcm-14-03829-s001.zip › jcm-3567302-supplementary.pdf]

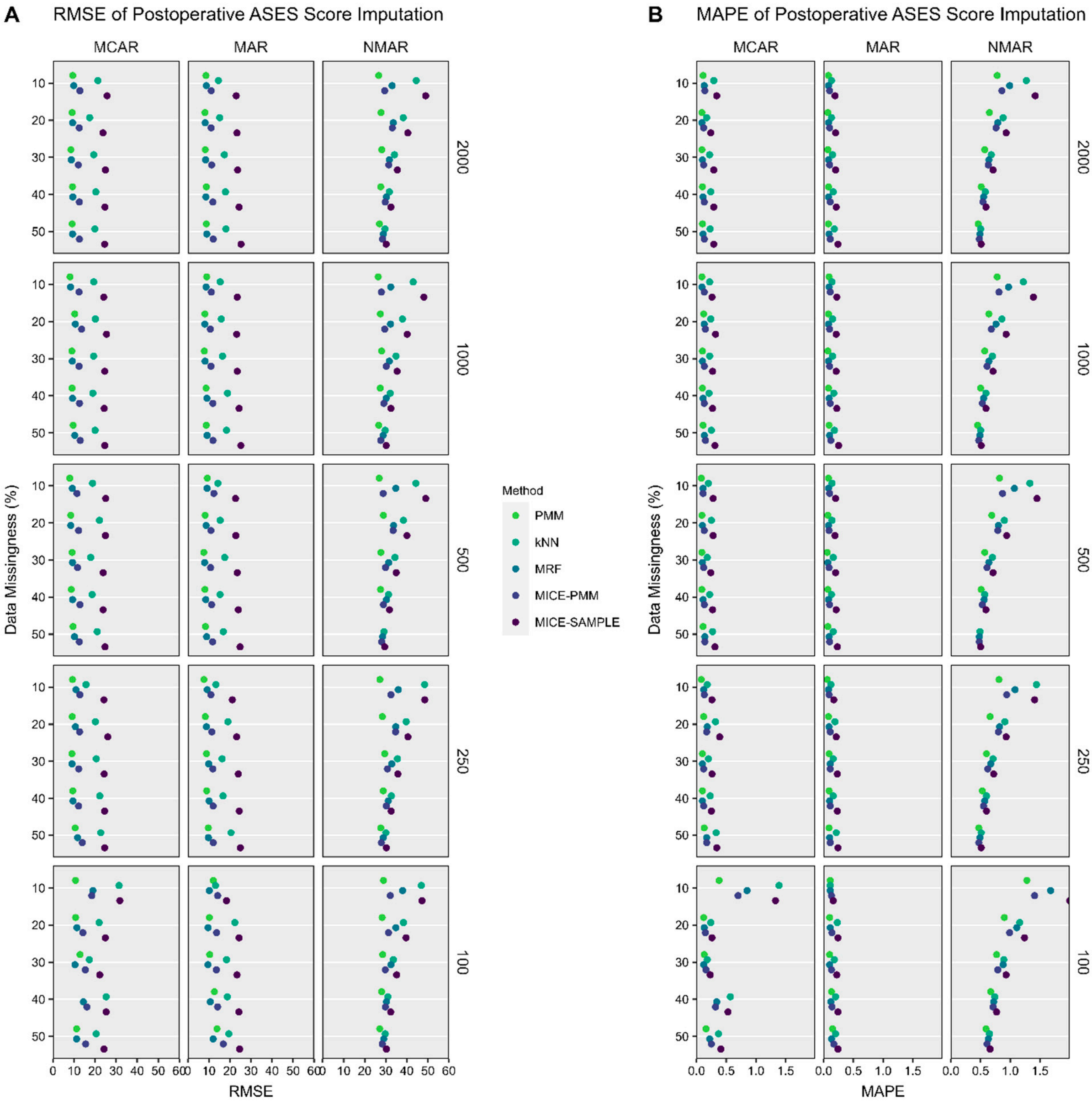

Supplementary Figure S1. RMSE and MAPE denoting the error between imputed values (with correlated scores available) and the original native data that was deleted. Single imputation with PMM and MRF and MICE with PMM performed well for MCAR and MAR conditions, whereas all methods performed poorly when data was NMAR. *ASES*, American Shoulder and Elbow Surgeons; *k-NN*, k-nearest neighbor; *MAPE*, mean absolute percentage error; *MAR*, missing at random; *MCAR*, missing completely at random; *MICE*, multiple imputation with chained equations; *MRF*, multivariate random forest; *NMAR*, not missing at random; *PMM*, predictive mean matching; *RMSE*, root mean squared error.

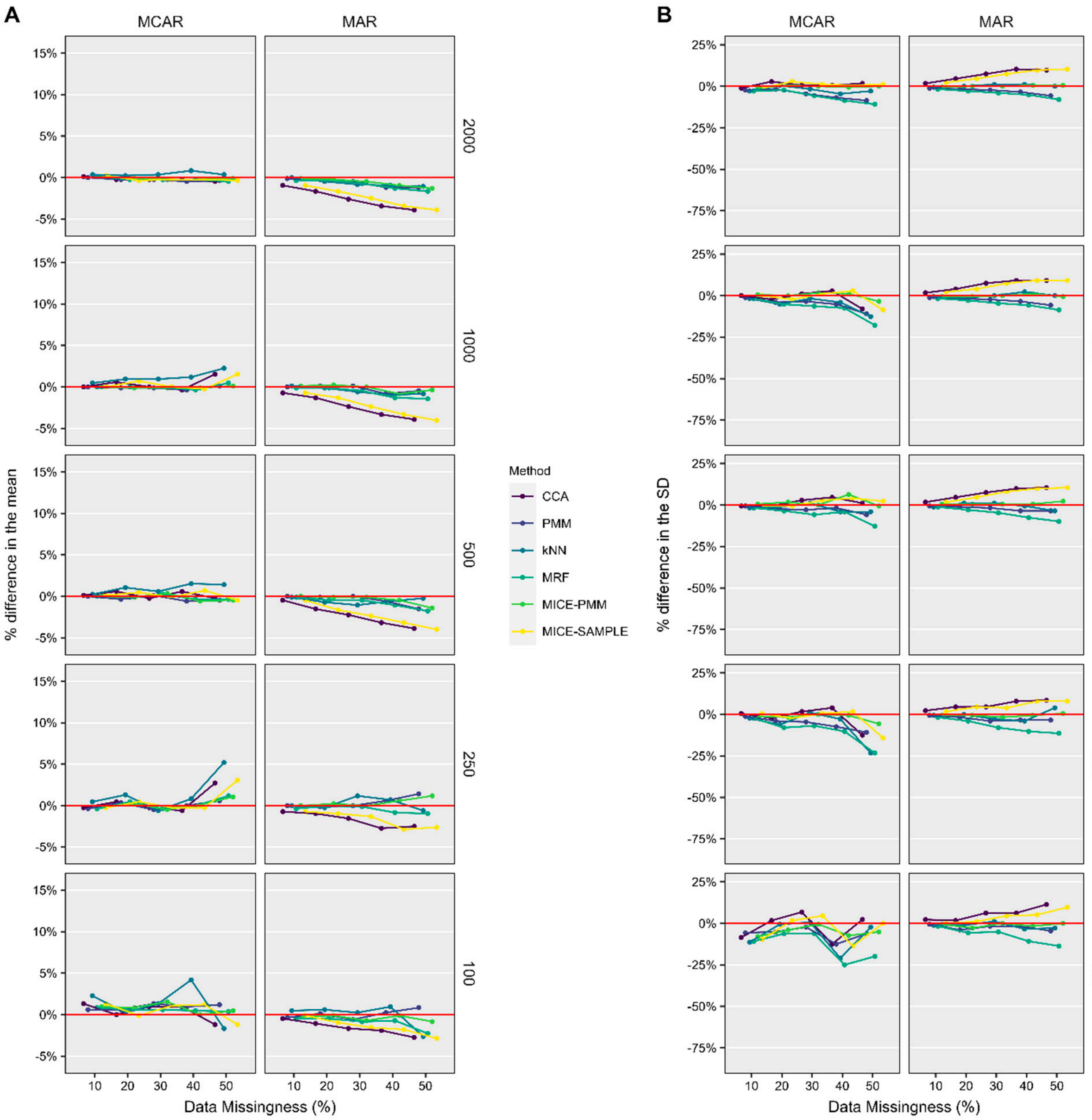

Supplementary Figure S2. Percent error in the mean (A) and standard deviation (B) of postoperative ASES scores (<u>with correlated scores available</u>) after missing data was remedied by each of the candidate methods compared to the native, complete dataset. *ASES*, American Shoulder and Elbow Surgeons; *k-NN*, k-nearest neighbor; *MAR*, missing at random; *MCAR*, missing completely at random; *MICE*, multiple imputation with chained equations; *MRF*, multivariate random forest; *NMAR*, not missing at random; *PMM*, predictive mean matching; *SD*, standard deviation.

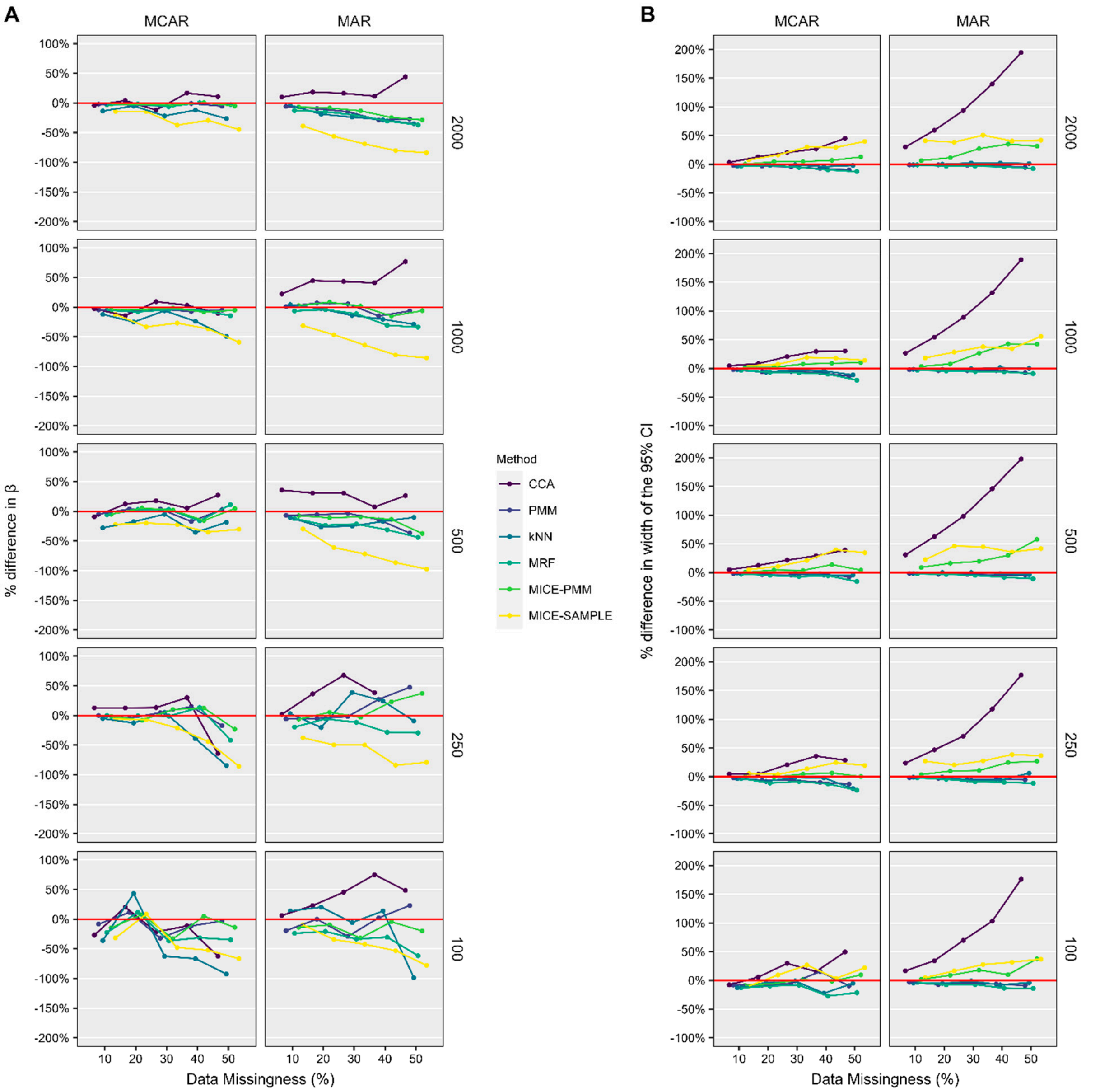

Supplementary Figure S3. Percent difference in the regression coefficient β (A) and the width of the 95% CI (B) for the relationship between preoperative and postoperative ASES score from multivariable linear regression performed on datasets after candidate methods were utilized to remedy missing data (<u>with correlated scores available</u>) compared to the native, complete dataset. *ASES*, American Shoulder and Elbow Surgeons; *CI*, confidence interval; *k-NN*, k-nearest neighbor; *MAR*, missing at random; *MCAR*, missing completely at random; *MICE*, multiple imputation with chained equations; *MRF*, multivariate random forest; *NMAR*, not missing at random; *PMM*, predictive mean matching.

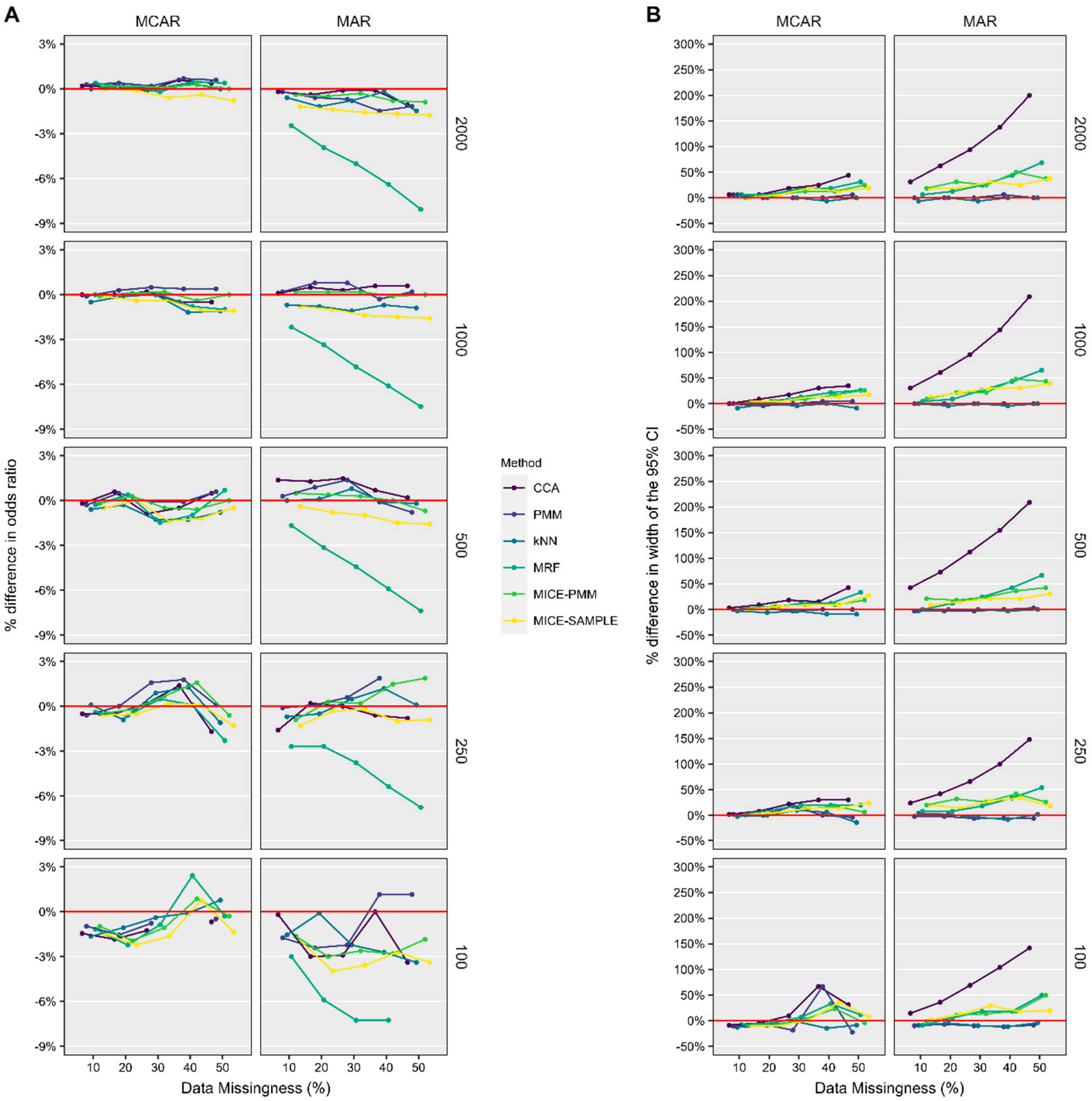

Supplementary Figure S4. Percent difference in the odds ratio (A) and the width of the 95% CI (B) for obtaining a 90th percentile ASES score from multivariable logistic regression performed on datasets after candidate methods were utilized to remedy missing data (with correlated scores available) compared to the native, complete dataset. *ASES*, American Shoulder and Elbow Surgeons; *CI*, confidence interval; *k-NN*, k-nearest neighbor; *MAR*, missing at random; *MCAR*, missing completely at random; *MICE*, multiple imputation with chained equations; *MRF*, multivariate random forest; *NMAR*, not missing at random; *PMM*, predictive mean matching.

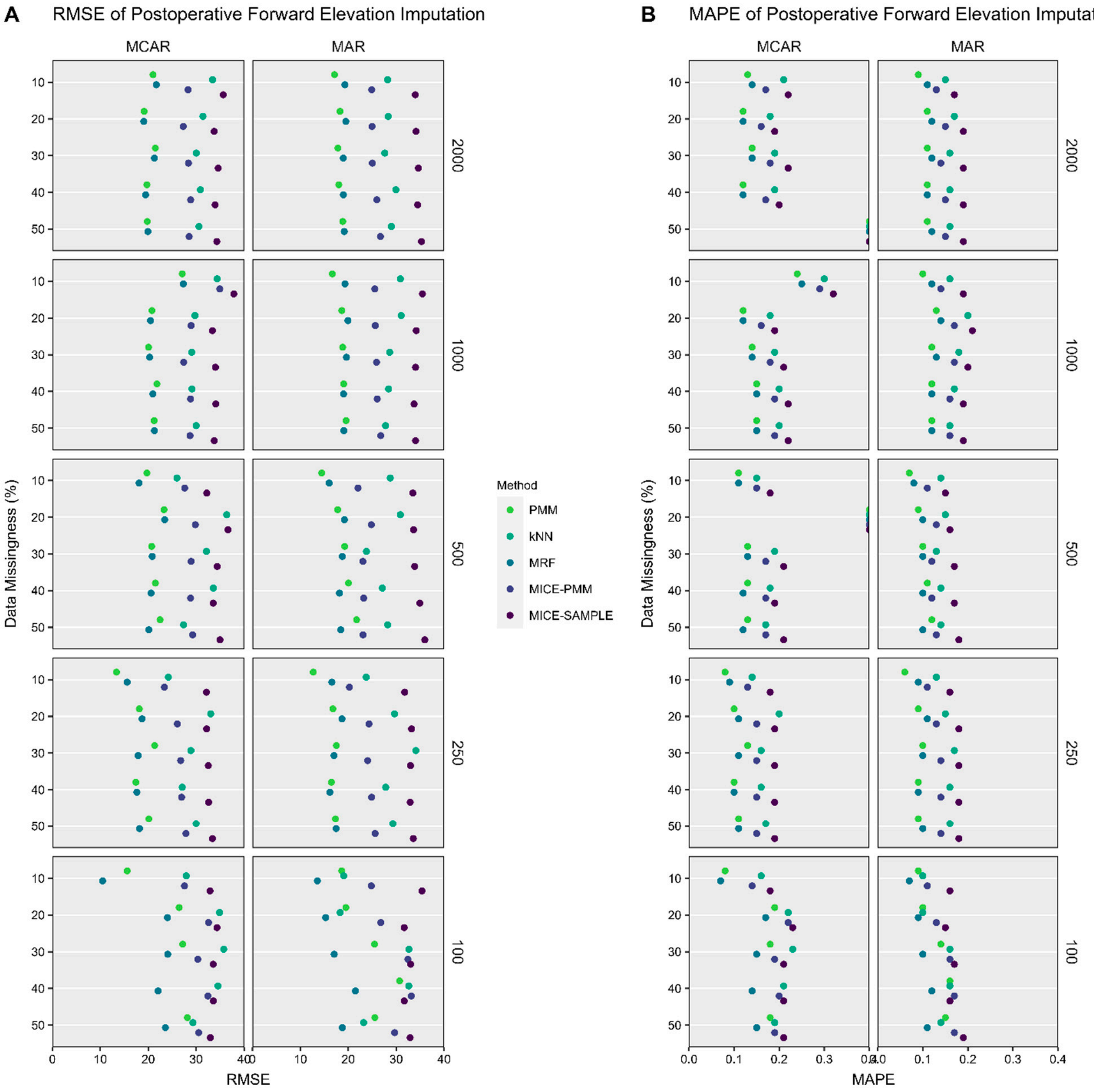

Supplementary Figure S5. RMSE and MAPE denoting the error between imputed <u>forward elevation</u> values and the original native data that was deleted. Single imputation with PMM and MRF and MICE with PMM performed well for MCAR and MAR conditions, whereas all methods performed poorly when data was NMAR. *k-NN*, k-nearest neighbor; *MAPE*, mean absolute percentage error; *MAR*, missing at random; *MCAR*, missing completely at random; *MICE*, multiple imputation with chained equations; *MRF*, multivariate random forest; *NMAR*, not missing at random; *PMM*, predictive mean matching; *RMSE*, root mean squared error.

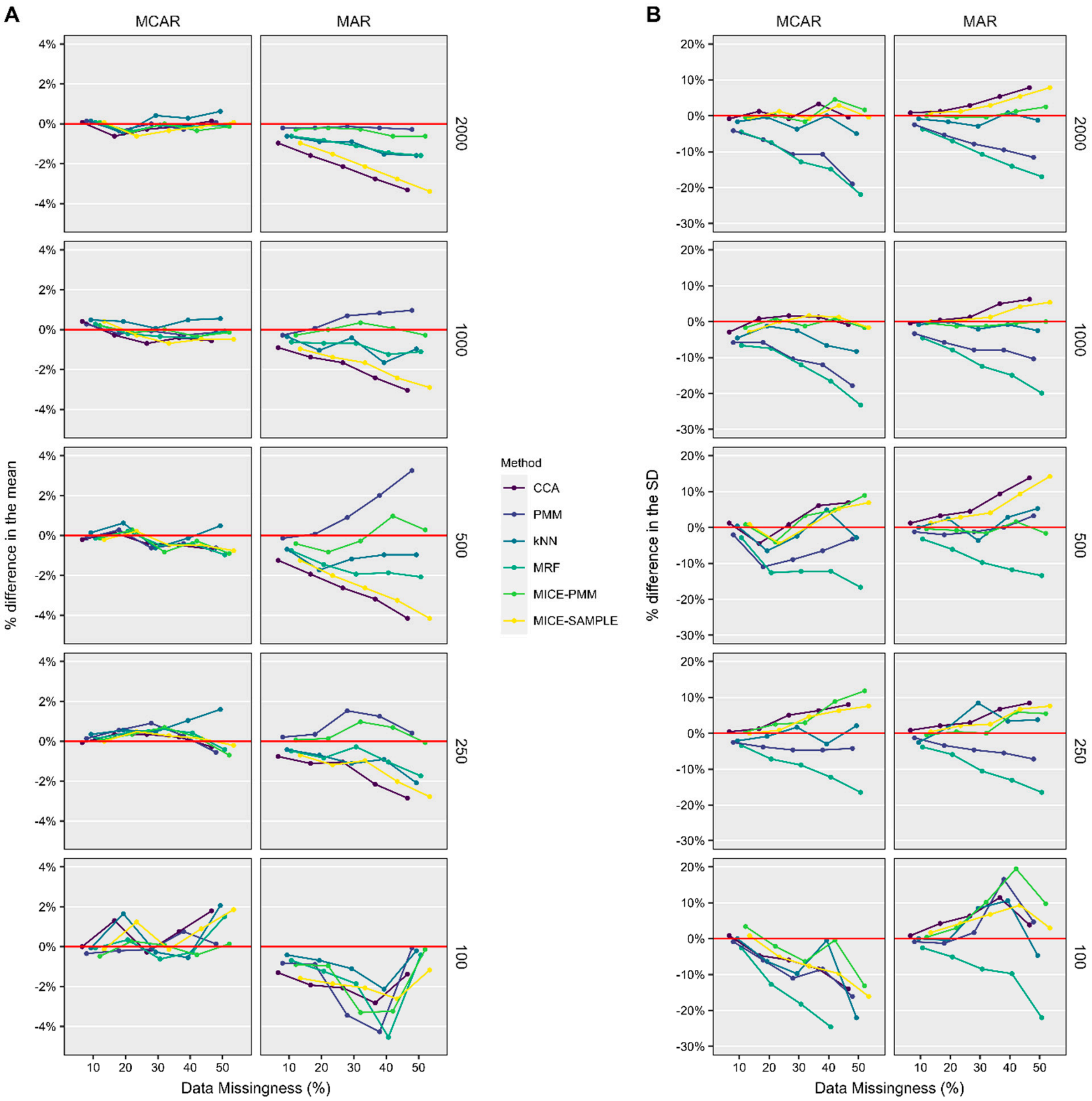

Supplementary Figure S6. Percent error in the mean (A) and standard deviation (B) of postoperative forward elevation after missing data was remedied by each of the candidate methods compared to the native, complete dataset. *ASES*, American Shoulder and Elbow Surgeons; *k-NN*, k-nearest neighbor; *MAR*, missing at random; *MCAR*, missing completely at random; *MICE*, multiple imputation with chained equations; *MRF*, multivariate random forest; *NMAR*, not missing at random; *PMM*, predictive mean matching; *SD*, standard deviation.

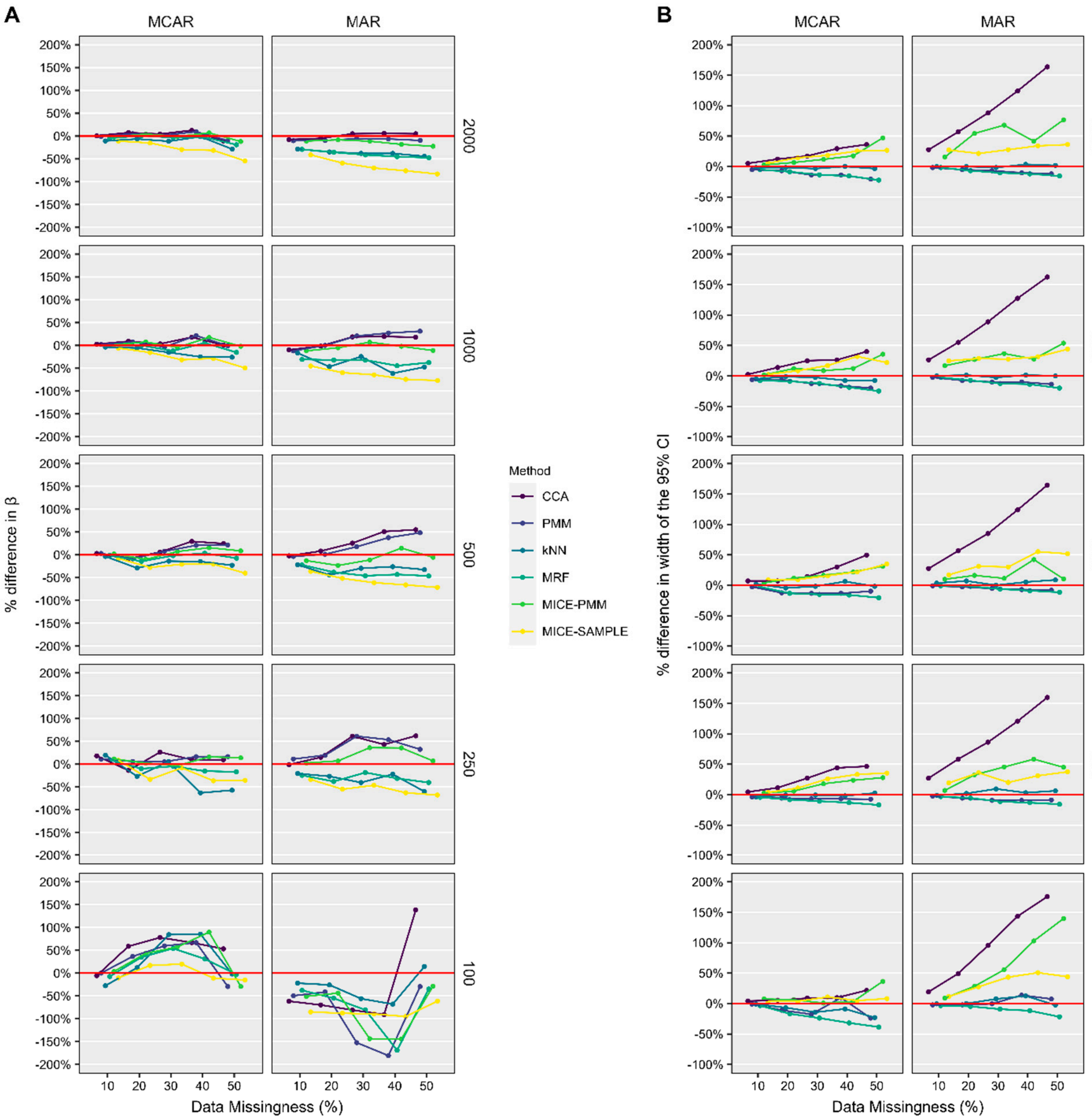

Supplementary Figure S7. Percent difference in the regression coefficient β (A) and the width of the 95% CI (B) for the relationship between preoperative and postoperative forward elevation from multivariable linear regression performed on datasets after candidate methods were utilized to remedy missing data compared to the native, complete dataset. *ASES*, American Shoulder and Elbow Surgeons; *CI*, confidence interval; *k-NN*, k-nearest neighbor; *MAR*, missing at random; *MCAR*, missing completely at random; *MICE*, multiple imputation with chained equations; *MRF*, multivariate random forest; *NMAR*, not missing at random; *PMM*, predictive mean matching.

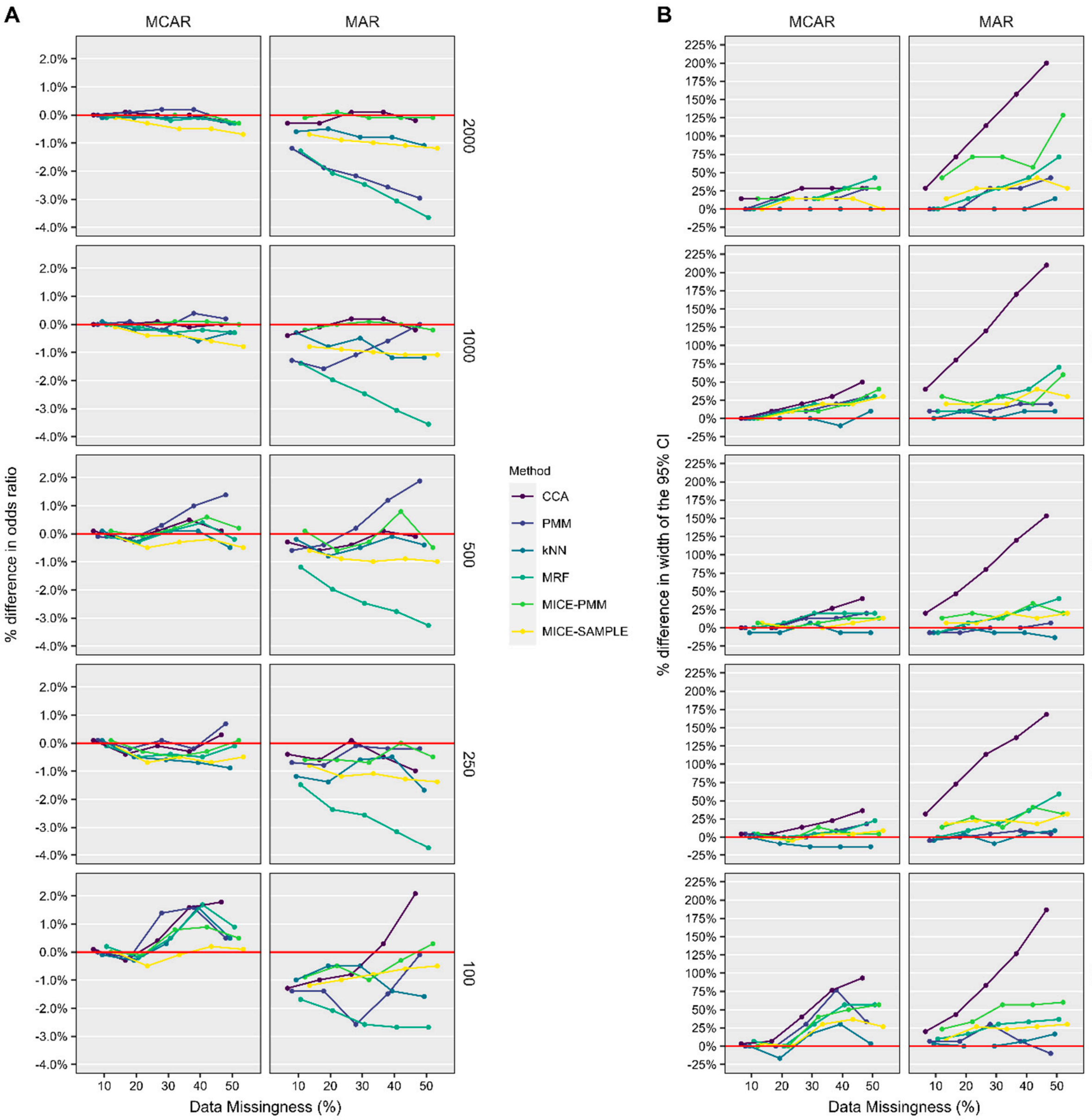

Supplementary Figure S8. Percent difference in the odds ratio (A) and the width of the 95% CI (B) for the relationship between the preoperative forward elevation and odds of achieving a 90th percentile postoperative forward elevation from multivariable logistic regression performed on datasets after candidate methods were performed to remedy missing data compared to the native, complete dataset.
